# Characterisation of a household norovirus outbreak occurred in Valencia (Spain)

**DOI:** 10.1186/s12879-016-1455-9

**Published:** 2016-03-12

**Authors:** Noelia Carmona-Vicente, Manuel Fernández-Jiménez, Susana Vila-Vicent, Jesús Rodríguez-Díaz, Javier Buesa

**Affiliations:** Department of Microbiology, School of Medicine, University of Valencia, Avda. Blasco Ibáñez 17, 46010 Valencia, Spain

**Keywords:** Norovirus, Susceptibility, Histo-blood group antigens, FUT2, Secretor, Virus-like particles, IgA, IgG, Binding assay

## Abstract

**Background:**

Human noroviruses (NoVs) are the main cause of non-bacterial gastroenteritis worldwide. Several studies have linked human susceptibility to NoVs with the expression of histo-blood group antigens (HBGAs). In January 2012, a NoV gastroenteritis outbreak affected a household in Valencia, Spain, and the personal susceptibility to NoV was investigated.

**Methods:**

To reach this aim 8 members of the affected household were recruited for this study and their secretor status, ABO and Lewis antigens were determined. NoV-specific saliva IgA and serum IgG antibody titers were analyzed. Their capacity to block viral binding to saliva receptors was analyzed, using virus-like particles (VLPs) of the NoV GII.4 genotype, 2006b variant, and saliva from a secretor O blood type donor.

**Results:**

The most relevant finding was that an asymptomatic non-secretor individual shed NoVs in his stools. Interestingly, anti-NoV IgA antibody titers in saliva from secretor and non-secretor individuals showed no differences. On the contrary, high titers of NoV-specific IgG antibody were found in both convalescent sera and in sera collected 1 year post-infection, but only from secretor individuals. NoV GII.4-2006b VLP binding to receptors present in the saliva was efficiently blocked only by sera from secretor positive individuals.

**Conclusions:**

Despite the small number of individuals involved in this outbreak, this study reinforces the idea that susceptibility to human NoV is both dependent on the HBGA profile of the individuals as well as on the viral genotype and variant. We also show that the immunity to NoV lasts for at least 1 year after infection, demonstrating that symptomatic infections strongly stimulate immune responses.

## Background

Noroviruses (NoVs), members of the *Caliciviridae* family, are small, positive-polarity RNA viruses and the most important cause of human viral gastroenteritis worldwide [1]. They are classified into 7 genogroups, although genogroups I (GI) and GII cause most human NoV infections [2]. These two genogroups are subdivided into 9 and 22 different genotypes, respectively. Several genotype GII.4 variants have emerged and caused the majority of all NoV-associated gastroenteritis outbreaks and sporadic infections over the past 2 decades [3]. It has been shown that there are differences in NoV susceptibility in humans [4]. This different susceptibility is associated to histo-blood group antigens (HBGAs) (i.e. the ABO blood group, the Lewis phenotype and the secretor status) [5] since NoVs recognize human histo-blood group antigens [6–8].

The expression of the α-1,2-fucosyltransferase (FucT II) enzyme, encoded by the *FUT2* gene, determines the secretor status of an individual [9]. Secretor positive individuals have A, B, H, Le^b^ and/or Le^y^ antigens. In contrast, secretor negative or non-secretor individuals have an inactive FUT2, leading to the lack of A, B, H, Le^b^ and/or Le^y^ antigens. However, they may be Le^a^ and Le^x^ positive as products of *FUT3* [10, 11]. The Lewis antigen is determined by the expression of the α-1,3-fucosyltransferase (FucT III) enzyme encoded by *FUT3*. Homozygous carriers of inactive *FUT3* alleles lack Le^a^ and Le^b^ structures, and such individuals are denoted Lewis-negative and constitute about 5 % of the Caucasian population. In addition to HBGAs, intestinal microbiota seems to have a role in susceptibility to NoV infection. It was recently demonstrated that human NoV infection can take place in vitro by using B cells as hosts and that this infectivity is linked to the intestinal microbiota [12]. In agreement with these findings, murine NoV (MNoV) infection in antibiotic-treated mice is reduced compared with mice with normal intestinal microbiota. Microbiota ablation with antibiotics also prevented persistent MNoV infection in mice [13].

It has been previously shown that human susceptibility to NoV is not completely determined by the secretor status, since secretor negative individuals can also be infected by NoVs [14, 15] spurring new studies to explore other host factors implied in NoV susceptibility.

In January 2012 a NoV gastroenteritis outbreak took place in a household in Valencia, Spain, that was a good opportunity to study the different individual susceptibility patterns to this viral infection.

## Methods

### Subjects and samples

Eight individuals were enrolled in this study, none of whom had taken any medication at or around the time of sample collection. The biosafety and ethics committees of the University of Valencia, Spain, approved the present study. Appropriate informed written consents were obtained from all individuals. Of these, at least 4 subjects were in contact with the suspected source of infection, a previously infected individual who was considered the index case, and three of them showed typical symptoms of NoV infection (nausea, vomiting, diarrhoea, abdominal pain and fever). The family members were living together and the outbreak was considered to be person-to-person transmitted. In order to determine the etiological agent of this outbreak, stool samples were collected from involved individuals with and without clinical symptoms. Serum samples from 3 household members were collected at 14 days (considered convalescent serum) and 1 year (considered memory serum) post-infection to analyze NoV-specific IgG antibodies and their blocking capacity. Blood samples were also collected to determine the histo-blood group antigens of each individual by hemagglutination. Saliva samples from all the household members were collected. The freshly collected saliva samples were centrifuged at 10,000 × g for 5 min to remove particulate material, host and microbial cells and boiled at 100 °C to inactivate antibodies. The supernatants were collected, divided into several aliquots and stored at -80 °C until their use. To minimize the effects of the circadian rhythm, saliva samples were consistently collected in the morning hours (between 8 and 10 a.m.). Also, participants were instructed not to smoke, eat, drink, or brush their teeth in the 2 h before saliva collection. For genotyping and NoV-specific IgA antibody detection, saliva was centrifuged, divided into several aliquots and stored directly at -80 °C without boiling to preserve IgA integrity.

### Norovirus detection

Viral RNA was extracted from 20 % stool suspensions in PBS using the Trizol LS reagent (Invitrogen, Paisley, Scotland), eluted in diethyl pyrocarbonate-treated water containing RNasin (Promega, Madison, WI) and stored at -80 °C. Reverse transcriptase-polymerase chain reaction (RT-PCR) for NoV was performed as described previously [16]. The genotype of NoV strains was determined by partial sequence analysis of the polymerase gene and the genotype was identified and classified using the typing tool at www.rivm.nl/mpf/norovirus/typingtool based on the similarity of the sequences to reference strains representing known genotypes [17].

### Secretor status analysis

The secretor (*FUT2*+) and non-secretor (*FUT2*-) status was investigated by genotyping the *FUT2* gene. Genomic DNA was extracted from saliva samples using the Qiagen QIAamp DNA Mini Kit. Genotyping for *FUT2* was performed by PCR-RFLP as described previously by Marionneau et al. [18]. A fragment of the FUT2 gene was amplified with primers 5′-GAGGAATACCGCCACATCCCGGGGGAGTAC-3′ (forward) and 5′-ATGGACCCCTACAAAGGTGCCCGGCCGGCT-3′ (reverse) and was digested with *Ava* II (Fermentas, Life Technologies, Alcobendas, Spain) for 2.5 h at 37 °C.

### ABO blood type and Lewis antigen determination

The presence of HBGAs was determined by detection of group A and B glycans by hemagglutination assay (ALBAclone® Monoclonal ABO Antisera, Alpha Laboratories, Eastleigh, England) and Lewis antigens were phenotyped by ELISA with monoclonal antibodies against Le^a^, Le^b^, Le^x^ and Le^y^ (Covance, Dedham, MA, USA). Briefly, plates were coated with saliva samples at 1/1,000 dilution in carbonate/bicarbonate buffer (pH 9.6) at 4 °C overnight. After blocking with PBS containing 3 % (w/v) bovine seroalbumin (PBS-BSA), plates were incubated with the different anti-Lewis antigen monoclonal antibodies (anti-Le^a^ BG-5, Le^b^ BG-6, Le^x^ BG-7 or Le^y^ BG-3, Covance) at 1/100 during 1 h at 37 °C and detected by a secondary mouse antibody mix (anti-IgG, anti-IgM and anti-IgA) (Sigma, Madrid, Spain) conjugated to horseradish peroxidase (HRP). After each step, plates were washed with PBS containing 0.05 % of Tween-20 (PBS-T). The reaction was developed by the addition of OPD Fast (Sigma) and stopped after 10 min incubation with 3 M H_2_SO_4_. Absorbance was measured at 492 nm in a microplate reader (Multiskan FC, Thermo Scientific, Alcobendas, Madrid).

### Norovirus VLPs

In the present study, VLPs corresponding to GII.4-Den Haag_2006b genotype were used. The recombinant baculovirus expressing the GII.4-2006b was already available in the laboratory [19]. The NoV VLPs were produced and purified as previously described [19]. Because VLPs from the GII.4-New Orleans_2009 variant causing the NoV gastroenteritis outbreak were not available for patients’ antibody testing, analyses were performed with VLPs of the GII.4-Den Haag_2006b variant, which share a 93.66 % homology at the amino acid level with the GII.4-2009 variant.

### Norovirus-specific IgA antibody in saliva samples

Microtiter plates were coated GII.4-2006b VLPs at 2 μg/ml in carbonate/bicarbonate buffer (pH 9.6) and incubated at 4 °C overnight. Plates were washed three times with PBS-T and blocked for 1 h at 37 °C with PBS-T 3 % BSA. After blocking, plates were incubated with serial dilutions of saliva samples (from 1/20 to 1/160) in PBS-T 1 % BSA for 1.5 h at 37 °C and NoV-specific IgA antibody was detected with a secondary anti-IgA human antibody conjugated with HRP (Sigma) at a dilution of 1/4,000 in PBS-T 1 % BSA for 1 h at 37 °C. As controls, all saliva samples were tested in parallel in wells coated with BSA at 2 μg/ml. To rule out nonspecific binding (false positives), boiled saliva samples were also used as negative controls on wells coated with VLPs. Wells were washed four times with PBS-T, and the bound antibody was detected by the addition of 50 μl of o-phenylenediamine (Sigma). The reaction was stopped at 10 min with 3 M H_2_SO_4_, and the absorbance was read at 492 nm (Multiskan FC spectrophotometer, Thermo Scientific).

### Norovirus-specific IgG antibody titers in serum samples

Purified GII.4-2006b VLPs were used to coat 96-well polystyrene microtiter plates as described above. After blocking with PBS-BSA 3 %, plates were incubated with serial serum dilutions from 1/200 to 1/12,800 diluted in PBS-T containing 1 % BSA for 1 h at 37 °C. Then HRP-conjugated anti-human IgG antibody (Santa Cruz Biotechnology) diluted 1/4,000 was added for 1 h and the reaction was developed and measured as described above. A sample was considered positive when its OD was three times higher than the absorbance value of the same sample in the specificity control (wells coated with 2 μg/ml of BSA). The titer of each sample was considered the inverse of the last dilution showing a positive reaction.

### Binding blocking assays

Microtiter plates were coated with saliva at 1/500 in carbonate/bicarbonate buffer (pH 9.6) overnight at 4 °C. Plates were washed three times with PBS-T and blocked for 1 h at 37 °C with PBS-T 3 % BSA. Plates were then incubated for 1.5 h with GII.4-2006b VLPs (2 μg/ml) that had been pre-incubated during 1 h at 37 °C in the presence of the different sera at serial 2-fold dilutions from 1/100 to 1/3.200, or with PBS in the negative control. The mixtures were then added to the saliva-coated plates and incubated for 1 h at 37 °C. The detection was performed with the anti-NoV rabbit polyclonal serum at 1/1,000 followed by a secondary HRP-conjugated anti-rabbit IgG antibody diluted at 1/2,000.

Blocking of VLP binding by the tested sera was determined by comparing the OD values obtained in wells in duplicate containing potential blocking reagents against the control wells (without the blocking steps). Blocking was considered when the OD value was less than 50 % of the positive control OD value (ranging from 0.8 to 1.2 OD_492_ units).

## Results

### Gastroenteritis outbreak was caused by norovirus GII.4-New Orleans_2009 variant

In January 2012 three members of a household in Valencia showed typical symptoms of acute gastroenteritis caused by NoV 24 h after having been in contact with the index case. Clinical symptoms were nausea, vomiting, diarrhoea and fever, which lasted approximately 48 h. One of the patients had to be taken care of dehydration at the emergency room.

RT-PCR assay performed on stool samples collected (*n* = 3) from two symptomatic patients and from one asymptomatic individual revealed the presence of NoV GII.4, specifically the GII.4-New Orleans_2009 variant. As no other enteric viruses (rotavirus or adenovirus) were detected by molecular analyses neither enteropathogenic bacteria were isolated, it was concluded that NoV was the etiological agent of this outbreak. The most relevant result was that the asymptomatic individual also shed NoVs as his faecal sample was also positive.

### Non-secretor individual (sese) showed no symptoms after norovirus GII.4-New Orleans_2009 infection

Twenty-five percent (2/8) of the household members were secretor homozygous (SeSe), 50 % (4/8) secretor heterozygous (Sese) and the remaining 25 % (2/8), non-secretor (sese). Secretor individuals showed a Le^a+^Le^b+^/Le^x-^Le^y+^ phenotype and non-secretor had the typical Lewis phenotype, Le^a+^Le^b-^/Le^x+^Le^y-^ (Table 1). Three individuals showed symptoms of which two were secretor homozygous (SeSe) and blood group A, and the other one was heterozygous (Sese) and blood group O. Non-secretor individuals showed no symptoms, while at least in one of them the infection was confirmed by RT-PCR.

**Table 1:** List of the individuals involved in the present study, specifying gender, age, ABO blood group, secretor status based on the *FUT2* gene analysis, Lewis phenotype and faecal NoV shedding detected by RT-PCR

Sample	Symptoms	Sex	Age	ABO blood group	FUT2	Lewis antigens	NoV RT-PCR
1	+	F	63	A	Se + Se+	Le^a+^Le^b+^/Le^x-^Le^y+^	+
2	-	M	64	A	se-se-	Le^a+^Le^b-^/Le^x+^Le^y-^	+
3	+	F	28	O	Se + se-	Le^a+^Le^b+^/Le^x-^Le^y+^	+
4	+	M	37	A	Se + se-	Le^a+^Le^b+^/Le^x-^Le^y+^	ND
5	-	F	8	A	se-se-	Le^a+^Le^b-^/Le^x+^Le^y-^	ND
6	-	M	34	O	Se + se-	Le^a+^Le^b+^/Le^x-^Le^y+^	ND
7	-	F	39	A	Se + se-	Le^a+^Le^b+^/Le^x-^Le^y+^	ND
8	-	M	47	B	Se + Se+	Le^a+^Le^b+^/Le^x-^Le^y+^	ND

*F* Female, *M* Male, *ND* not determined

### Titers of salivary anti-norovirus IgA antibodies

**Fig. 1:**
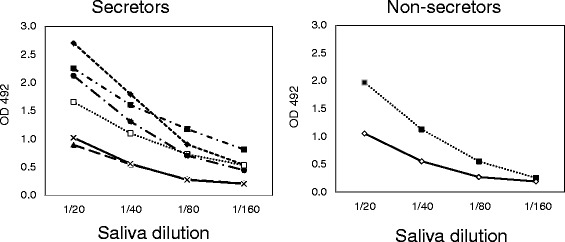
NoV-specific IgA antibody levels. IgA antibody levels against GII.4-Den Haag_2006b NoV VLPs in saliva samples from 6 secretor (*left*) and 2 non-secretor (*right*) individuals. The ELISA plates were coated with the NoV VLPs and serial dilutions of saliva samples (1/20 to 1/160) were tested for NoV-specific IgA

Salivary IgA antibodies to the GII.4-2006b were positive in all samples. Although the number of samples was too small to perform statistical analyses, no differences were found in secretory IgA levels from the two analyzed groups: secretors and non-secretors (Fig. 1). These results suggest that non-secretors are also susceptible to NoV infection even if they do not show clinical symptoms, as it was also confirmed by RT-PCR.

### Norovirus-specific IgG antibody titers

Three sera were collected and compared with a convalescent serum from a sporadic case of NoV infection, which was used as the positive control. Of these sera, two were from secretor individuals that showed symptoms and the other one was from an infected non-secretor without symptoms.

**Fig. 2:**
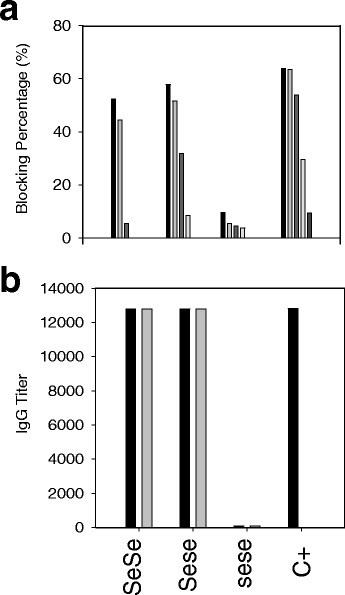
NoV-specific IgG antibody titers and blocking activity of sera from members of a household suffering an acute gastroenteritis outbreak. ELISA plates were coated with NoV VLPs from GII.4-Den Haag_2006b variant to determine the antibody titer according to the secretor (Sese or SeSe) or non-secretor (sese) status. The positive control corresponds to a convalescent serum from another independent gastroenteritis sporadic case. **a** The upper panel of the figure represents the blocking percentage of the convalescent sera (14 days), from secretors (SeSe and Sese) and non-secretors (sese), that were tested at serial dilutions 1/100, 1/200, 1/400, 1/800 and 1/1,600 (*black to clear grey*). A percentage higher of 50 % of inhibition was considered as blocking activity. **b** The lower panel shows the IgG titer against GII.4-Den Haag_2006b VLPs at 2 different times: 14 days (*black*) and 1 year (*grey*)

All tested convalescent serum samples showed antibody titers higher than 1/100 to the GII.4-2006b variant. IgG serum titers showed significant differences (*P* < 0.01) between secretors and the non-secretor (Fig. 2). Titers of NoV-specific IgG antibody of 1/12,800 were the highest titers found, corresponding to the two symptomatic infected individuals in their convalescence (14 days p.i.) and memory (1 year p.i.) serum samples, independently whether they were homo- or heterozygous for the FUT2 mutation. The serum from the non-secretor individual showed no changes in its antibody titers, despite having been in contact with the virus and showing the lowest antibody titer (Fig. 2).

### Blockade of the binding of VLPs to saliva

We examined the blocking capacity of the different serum samples (both convalescent and memory sera) on the binding of GII.4-2006b VLPs to saliva from a secretor, blood type O individual. As expected, the binding to receptors present in the saliva was blocked efficiently by secretor individual sera, up to 60 % (Fig. 2). On the other hand, the non-secretor serum sample did not block the binding at all. The results obtained with the memory serum showed the same blocking rate as the corresponding convalescent serum samples.

## Discussion

Recent studies have shown that host genetic factors such as the *FUT2* and *FUT3* genotypes strongly affect human susceptibility to NoVs [20]. Due to the lack of a good animal or in vitro model to investigate NoV pathogenesis, evidences in this field have been obtained both from volunteers studies and from outbreaks of natural infections [5], like the outbreak here reported.

In this outbreak occurred in Valencia in 2012, although few individuals were affected, different susceptibility to NoV diarrhoea in secretor-positive and secretor-negative individuals was observed, reinforcing the idea that non-secretors are naturally protected to NoV infections [21]. Surprisingly, one of the stool samples positive for NoV by RT-PCR was from a secretor-negative individual. This result is highly relevant since it indicates that NoV replication occurred in the non-secretor individual but without causing symptoms. This observation was confirmed with the finding of a high anti-NoV IgA titer in the saliva of this non-secretor individual, similar to the titers in secretor-positive patients, thus showing that infection had indeed occurred. In the past we also described a non-secretor that was susceptible to NoV GII.4-Hunter_2004 NoV infection, but in that case symptoms were also present [14]. In order to become symptomatically infected (2004 outbreak) or asymptomatically (2012 outbreak) non-secretors must display receptors for NoVs, raising the question of what other receptors, not yet known, may be implicated in NoV infections. A recent study showed that NoV VLPs did not colocalize with H or Lewis antigens in epithelial cells, reinforcing the idea that other receptors are involved in NoV infections [22]. Gangliosids have also shown to bind NoVs [23]. In addition, enteric bacteria also seem to play a role in NoV infections acting themselves as co-receptors [12, 24, 25]. Another interesting result is that the secretor-negative individual did not develop IgG antibodies against NoV, in contrast to what it was observed with the salivary IgA. It can be argued that the absence of symptoms could be due to a lower infectivity of the virus in the secretor-negative individual, in whom it only elicited a mucosal immune response. On the other hand, high IgG titers were found in serum samples from infected secretors at the outbreak. Seroconversion occurred 2 weeks after the infection and the IgG titers did not decrease 1 year after infection. These results are relevant, since it has been reported that previous infections do not protect against subsequent NoV infections [26]. Our results suggest that this lack of protection might be due to viral evolution [3, 27–29] instead of a low immune stimulation. In fact, several seroepidemiologic studies of NoV-specific antibodies in different populations have been performed [19, 30, 31], showing high seroprevalence levels and consequently a good immunogenicity of NoVs. The blocking assays showed that serum IgG antibodies were able to block the attachment of VLPs to saliva, reinforcing the idea that NoV infections elicit protective immunity.

## Conclusions

Although the number of individuals involved in this outbreak was low, limiting the possibility of drawing conclusions, our results confirm the hypothesis that susceptibility to human noroviruses is both dependent on secretor status of the individuals and on the infecting NoV strain. We also have shown that the immunity to NoV lasts for at least 1 year after infection, confirming that symptomatic infection strongly stimulates an antibody response.

## Abbreviations

NoVs: Noroviruses
HBGAs: Histo-blood group antigens
VLPs: Virus-like particles
RT-PCR: Reverse transcriptase-polymerase chain reaction
FucT II: α-1,2-fucosyltransferase
FucT III: α-1,3-fucosyltransferase
MNoV: Murine norovirus
